# The power of TOPMed imputation for the discovery of Latino-enriched rare variants associated with type 2 diabetes

**DOI:** 10.1007/s00125-023-05912-9

**Published:** 2023-05-06

**Authors:** Alicia Huerta-Chagoya, Philip Schroeder, Ravi Mandla, Aaron J. Deutsch, Wanying Zhu, Lauren Petty, Xiaoyan Yi, Joanne B. Cole, Miriam S. Udler, Peter Dornbos, Bianca Porneala, Daniel DiCorpo, Ching-Ti Liu, Josephine H. Li, Lukasz Szczerbiński, Varinderpal Kaur, Joohyun Kim, Yingchang Lu, Alicia Martin, Decio L. Eizirik, Piero Marchetti, Lorella Marselli, Ling Chen, Shylaja Srinivasan, Jennifer Todd, Jason Flannick, Rose Gubitosi-Klug, Lynne Levitsky, Rachana Shah, Megan Kelsey, Brian Burke, Dana M. Dabelea, Jasmin Divers, Santica Marcovina, Lauren Stalbow, Ruth J. F. Loos, Burcu F. Darst, Charles Kooperberg, Laura M. Raffield, Christopher Haiman, Quan Sun, Joseph B. McCormick, Susan P. Fisher-Hoch, Maria L. Ordoñez, James Meigs, Leslie J. Baier, Clicerio González-Villalpando, Maria Elena González-Villalpando, Lorena Orozco, Lourdes García-García, Andrés Moreno-Estrada, Mexican Biobank, Carlos A. Aguilar-Salinas, Teresa Tusié, Josée Dupuis, Maggie C. Y. Ng, Alisa Manning, Heather M. Highland, Miriam Cnop, Robert Hanson, Jennifer Below, Jose C. Florez, Aaron Leong, Josep M. Mercader

**Affiliations:** 1 Programs in Metabolism and Medical & Population Genetics, Broad Institute of Harvard and MIT, Cambridge, MA USA; 2 Center for Genomic Medicine, Massachusetts General Hospital, Boston, MA USA; 3 Departamento de Medicina Genómica y Toxicología Ambiental, Instituto de Investigaciones Biomédicas, Universidad Nacional Autónoma de México, Mexico City, Mexico; 4 Unidad de Biología Molecular y Medicina Genómica, Instituto Nacional de Ciencias Médicas y Nutrición, Mexico City, Mexico; 5 Diabetes Unit, Massachusetts General Hospital, Boston, MA USA; 6 Department of Medicine, Massachusetts General Hospital, Boston, MA USA; 7 Vanderbilt Genetics Institute, Vanderbilt University Medical Center, Nashville, TN USA; 8 ULB Center for Diabetes Research, Medical Faculty, Université Libre de Bruxelles, Brussels, Belgium; 9 Department of Medicine, Harvard Medical School, Boston, MA USA; 10 Division of Endocrinology, Boston Children’s Hospital, Boston, MA USA; 11 Department of Biomedical Informatics, University of Colorado School of Medicine, Aurora, CO USA; 12 Division of General Internal Medicine, Massachusetts General Hospital, Boston, MA USA; 13 Department of Biostatistics, Boston University School of Public Health, Boston, MA USA; 14 Department of Endocrinology, Diabetology and Internal Medicine, Medical University of Bialystok, Bialystok, Poland; 15 Clinical Research Centre, Medical University of Bialystok, Bialystok, Poland; 16 Vanderbilt Genetics Institute, Division of Genetic Medicine, Vanderbilt University Medical Center, Nashville, TN USA; 17 Analytic and Translational Genetics Unit, Massachusetts General Hospital, Boston, MA USA; 18 WELBIO, Université Libre de Bruxelles, Brussels, Belgium; 19 Department of Clinical and Experimental Medicine, and AOUP Cisanello University Hospital, University of Pisa, Pisa, Italy; 20 Department of Pediatrics, University of California San Francisco, San Francisco, CA USA; 21 Department of Pediatrics, University of Vermont, Burlington, VT USA; 22 Department of Pediatrics, Boston Children’s Hospital, Boston, MA USA; 23 Pediatric Endocrinology, Diabetes, and Metabolism, Case Western Reserve University and Rainbow Babies and Children’s Hospital, Cleveland, OH USA; 24 Department of Pediatrics, Division of Pediatric Endocrinology and Pediatric Diabetes Center, Massachusetts General Hospital, Boston, MA USA; 25 Pediatric Endocrinology and Diabetes, Children’s Hospital of Philadelphia, Philadelphia, PA USA; 26 Pediatric Endocrinology, University of Colorado School of Medicine, Aurora, CO USA; 27 Biostatistics Center, The George Washington University, Rockville, MD USA; 28 Department of Epidemiology, University of Colorado School of Medicine, Aurora, CO USA; 29 NYU Langone Health, New York, NY USA; 30 Medpace Reference Laboratories, Cincinnati, OH USA; 31 The Charles Bronfman Institute of Personalized Medicine, Icahn School of Medicine at Mount Sinai, New York, NY USA; 32 Novo Nordisk Foundation Center for Basic Metabolic Research, Faculty of Health and Medical Science, University of Copenhagen, Copenhagen, Denmark; 33 Division of Public Health Science, Fred Hutchinson Cancer Center, Seattle, WA USA; 34 Department of Genetics, University of North Carolina at Chapel Hill, Chapel Hill, NC USA; 35 Department of Population and Public Health Sciences, University of Southern California, Los Angeles, CA USA; 36 Norris Comprehensive Cancer Center, University of Southern California, Los Angeles, CA USA; 37 Department of Biostatistics, University of North Carolina at Chapel Hill, Chapel Hill, NC USA; 38 School of Public Health, The University of Texas Health Science Center at Houston, Brownsville, TX USA; 39 Unidad de Biología Molecular y Medicina Genómica, Instituto Nacional de Ciencias Médicas y Nutrición, Mexico City, Mexico; 40 Phoenix Epidemiology and Clinical Research Branch, National Institute of Diabetes and Digestive and Kidney Diseases, National Institutes of Health, Phoenix, AZ USA; 41 Centro de Estudios en Diabetes, Unidad de Investigacion en Diabetes y Riesgo Cardiovascular, Centro de Investigacion en Salud Poblacional, Instituto Nacional de Salud Pública, Mexico City, Mexico; 42 Laboratorio Inmunogénomica y Enfermedades Metabólicas, Instituto Nacional de Medicina Genómica, Mexico City, Mexico; 43 Instituto Nacional de Salud Pública, Cuernavaca, Mexico; 44 Laboratorio Nacional de Genómica para la Biodiversidad (LANGEBIO), Unidad de Genómica Avanzada (UGA), CINVESTAV, Irapuato, Mexico; 45 Unidad de Investigación de Enfermedades Metabólicas y Dirección de Nutrición, Instituto Nacional de Ciencias Médicas y Nutrición Salvador Zubirán, Mexico City, Mexico; 46 Clinical and Translational Epidemiology Unit, Massachusetts General Hospital, Boston, MA USA; 47 Department of Epidemiology, University of North Carolina at Chapel Hill, Chapel Hill, NC USA; 48 Division of Endocrinology, Erasmus Hospital, Université Libre de Bruxelles, Brussels, Belgium; 49 Diabetes Epidemiology and Clinical Research Section, National Institute of Diabetes and Digestive and Kidney Diseases, Phoenix, AZ USA; 50 Endocrine Division, Massachusetts General Hospital, Boston, MA USA

**Keywords:** GWAS meta-analysis, Latino population, Polygenic score, TOPMed imputation, Type 2 diabetes

## Abstract

**Aims/hypothesis:**

The Latino population has been systematically underrepresented in large-scale genetic analyses, and previous studies have relied on the imputation of ungenotyped variants based on the 1000 Genomes (1000G) imputation panel, which results in suboptimal capture of low-frequency or Latino-enriched variants. The National Heart, Lung, and Blood Institute (NHLBI) Trans-Omics for Precision Medicine (TOPMed) released the largest multi-ancestry genotype reference panel representing a unique opportunity to analyse rare genetic variations in the Latino population. We hypothesise that a more comprehensive analysis of low/rare variation using the TOPMed panel would improve our knowledge of the genetics of type 2 diabetes in the Latino population.

**Methods:**

We evaluated the TOPMed imputation performance using genotyping array and whole-exome sequence data in six Latino cohorts. To evaluate the ability of TOPMed imputation to increase the number of identified loci, we performed a Latino type 2 diabetes genome-wide association study (GWAS) meta-analysis in 8150 individuals with type 2 diabetes and 10,735 control individuals and replicated the results in six additional cohorts including whole-genome sequence data from the All of Us cohort.

**Results:**

Compared with imputation with 1000G, the TOPMed panel improved the identification of rare and low-frequency variants. We identified 26 genome-wide significant signals including a novel variant (minor allele frequency 1.7%; OR 1.37, *p*=3.4 × 10^−9^). A Latino-tailored polygenic score constructed from our data and GWAS data from East Asian and European populations improved the prediction accuracy in a Latino target dataset, explaining up to 7.6% of the type 2 diabetes risk variance.

**Conclusions/interpretation:**

Our results demonstrate the utility of TOPMed imputation for identifying low-frequency variants in understudied populations, leading to the discovery of novel disease associations and the improvement of polygenic scores.

**Data availability:**

Full summary statistics are available through the Common Metabolic Diseases Knowledge Portal (https://t2d.hugeamp.org/downloads.html) and through the GWAS catalog (https://www.ebi.ac.uk/gwas/, accession ID: GCST90255648). Polygenic score (PS) weights for each ancestry are available via the PGS catalog (https://www.pgscatalog.org, publication ID: PGP000445, scores IDs: PGS003443, PGS003444 and PGS003445).

**Graphical abstract:**

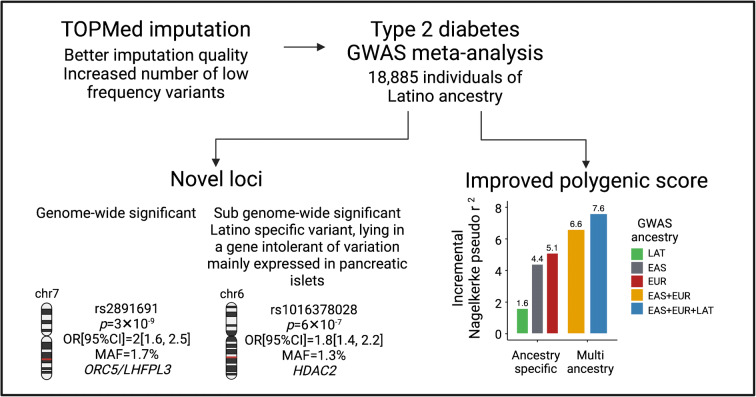

**Supplementary Information:**

The online version of this article (10.1007/s00125-023-05912-9) contains peer-reviewed but unedited supplementary material.

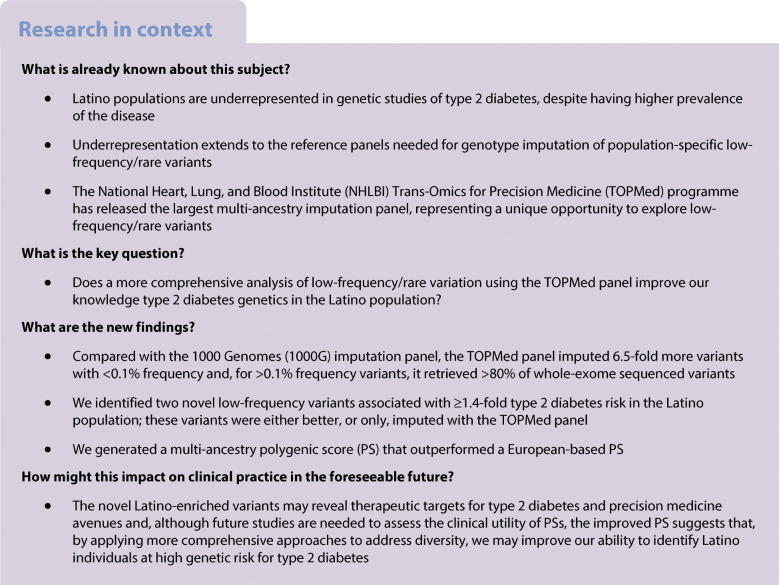



## Introduction

Latino is a diverse ethnic group recently admixed from Native American, European and African ancestries, with a high prevalence of metabolic disorders including type 2 diabetes. Although genetic studies in the Latino population are limited, they have revealed unexpected pathways and potential therapeutic targets for type 2 diabetes [1–4]. This is the case for a Native American haplotype within the *SLC16A11* gene identified as the main genetic contributor to type 2 diabetes in the Latino population [1, 4], a rare risk variant within *HNF1A* unique to Latino population [2] and a loss-of-function (LoF) Latino-enriched variant within *IGF2* associated with a 22% decrease in the odds of type 2 diabetes in heterozygous carriers [3].

Unlike genetically homogenous populations, the complex linkage disequilibrium (LD) structure of admixed populations imposes challenges in implementing statistical methods that are crucial to maximise genetic discoveries [5]. This is especially relevant for genotype imputation, a method used to estimate the genotype probabilities at genetic variants that have not been experimentally genotyped [6]. A major factor limiting the accuracy of genotype imputation in Latino samples has been the poor representation of their haplotypes in the reference panels (i.e. 352 from the latest version of the 1000 Genomes [1000G] imputation model) [7]. The multi-ancestry National Heart, Lung, and Blood Institute (NHLBI) Trans-Omics for Precision Medicine (TOPMed) programme has released a reference panel for genotype imputation that includes the highest sequencing coverage (i.e. 30×) and the largest number of reference samples (i.e. 97,256) to date, of which ~15% are from Latino individuals. It has been shown to increase the number of well-imputed low-frequency variants in the Hispanic Community Health Study/Study of Latinos (HCHS/SOL) [8, 9].

We hypothesised that by boosting the identification of variants in Latino samples with the recently released TOPMed reference panel, we would improve our knowledge of the genetic architecture of type 2 diabetes in the Latino population. The 1000G (1000G) panel was chosen as a comparison, since, besides TOPMed, it has the largest number of Latino samples. We performed a type 2 diabetes genome-wide association study (GWAS) meta-analysis, as well as association analyses on a collection of related phenotypes from TOPMed Latino imputed datasets to allow the interpretation of our novel variants that had low frequencies or were absent in other publicly available biobanks that mainly contained individuals of European ancestry. Finally, we leveraged the generated GWAS data to develop, in combination with GWAS data from other ancestries, a type 2 diabetes polygenic score (PS) for the Latino population.

## Methods

Detailed descriptions of the methods are given in electronic supplementary material (ESM) Methods.

### Discovery sample

**Fig. 1:**
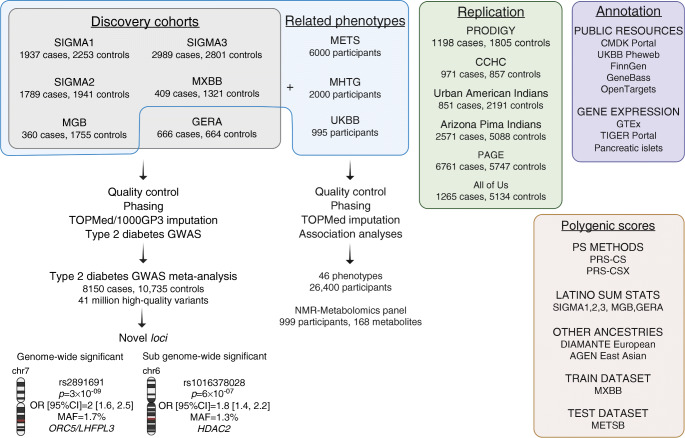
General overview of the study. Six cohorts of admixed Latino ancestry, representing a total of 8150 type 2 diabetes cases and 10,735 controls, were imputed with the TOPMed and 1000G Phase 3 panels (grey box). A type 2 diabetes GWAS meta-analysis of the imputed variants resulted in the identification of two novel loci, which were tested for replication in six additional Latino cohorts (green box). They were also interrogated for association with a collection of phenotypes in eight Latino cohorts (blue box) and for functional evidence in multiple available resources (purple box). The generated Latino type 2 diabetes GWAS data were used, in combination with GWAS from other ancestries, to construct ancestry-specific and multi-ancestry type 2 diabetes PSs (brown box). CMDK, Common Metabolic Disease Knowledge; sum stats, summary statistics

We aggregated data from six Latino cohorts with a sample size of 18,885 individuals (8150 with type 2 diabetes [cases] and 10,735 without [controls]): the Slim Initiative for Genomic Medicine in the Americas (SIGMA) [1–3]; the Mexican Biobank (MXBB) [10]; the Mass General Brigham (MGB) Biobank [11]; and the Genetic Epidemiology Research on Aging (GERA) [12] (Fig. 1 and ESM Table 1). We selected Latino samples based on their genetically estimated ancestry using principal components (PCs) and Admixture v1.3.0 [13] (ESM Fig. 1). All human research was approved by the relevant Institutional Review Boards and conducted according to the Declaration of Helsinki. All participants provided written informed consent.

### Genotyping and imputation

Genotyping was done using several commercially available genome-wide arrays, and for a subset of the samples (*N*=9520), we integrated whole-exome sequencing (WES) (ESM Table 1). We applied pre-imputation quality control to each dataset separately. Clean datasets were phased using SHAPEIT2 v2 [14]. For comparison purposes, we imputed the phased haplotypes using both 1000G Phase3 version 5 [15] and TOPMed reference panels freeze 8 [8].

### Imputation performance evaluation

**Fig. 2:**
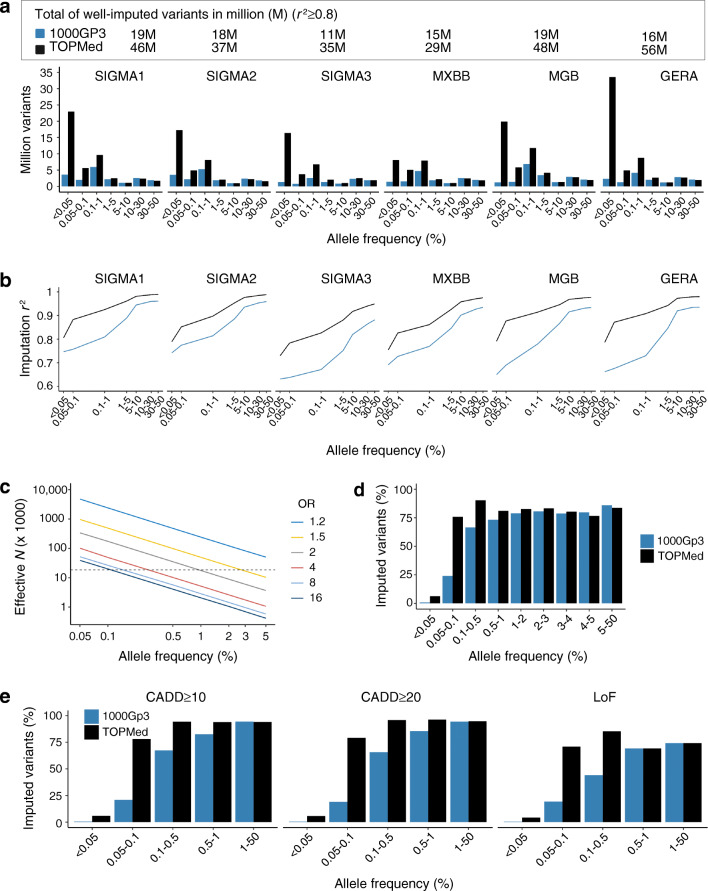
Performance of the TOPMed reference panel for the imputation of Latino samples. (**a**) Number of chromosome-wide well-imputed variants (imputation *r*^2^≥0.8) by AF for each analysed cohort when using the 1000GP3 (blue) or the TOPMed (black) reference panels. (**b**) Average chromosome-wide imputation quality by AF for each analysed cohort when using the 1000GP3 (blue) or the TOPMed (black) reference panels. (**c**) Effective sample size required for reaching 80% statistical power to detect genome-wide significant signals at different effect sizes (OR). The dotted lines show the discovery effective sample size of this study. (**d**) Percentage of the exome sequenced variants in chromosome 22 that could be imputed when using the 1000GP3 (blue) or the TOPMed (black) reference panels. (**e**) Percentage of the exome sequenced LoF and deleterious predicted variants based on CADD score in chromosome 22 that could be imputed when using the 1000GP3 (blue) or the TOPMed (black) reference panels

We evaluated the performance of TOPMed and 1000G imputations by summarising the chromosome-wise *r*^2^ quality measure and the number of well-imputed (*r*^2^≥0.8) variants at different allele frequency (AF) thresholds. We used available WES data from the SIGMA3 cohort and estimated the proportion of the sequenced variants in chromosome 22 that were well-imputed with TOPMed and 1000G panels at different WES AF thresholds. We used SnpEff v4.3 [16] to annotate the WES variants. We calculated the effective sample size (Neff) needed to reach 80% statistical power to detect genome-wide significant associations (α=5 × 10^–8^) at different effect sizes and AFs covered by the imputations (Fig. 2c).

### Type 2 diabetes GWAS meta-analysis

Association analyses were performed in each cohort with SNPTEST v2.5.4 [17]. Models were adjusted for sex, age, BMI and ten PCs to account for population structure. We ran additional models without adjusting for BMI. Only well-imputed variants (*r*^2^≥0.5) were meta-analysed using the inverse of the corresponding squared SEs in METALv2011-03-25 [18] We used a standard GWAS significance threshold of *p*<5 × 10^−8^.

We performed LD-based clumping on the genome-wide significant variants to keep one representative variant per region of LD. If the lead SNP lay within a previously reported type 2 diabetes locus, we defined it as conditionally distinct if showing evidence of residual association (*p*<5 × 10^−5^) after conditioning on each of the reported variants.

Variants with sub-genome-wide significance (*p*<1 × 10^−6^) that were only imputed with the TOPMed panel, showed increased frequency in the Latino population and were >250 kb from other reported genome-wide significant variants from European or East Asian ancestry large consortia [19, 20] were considered for further investigation.

### Replication sample

Variants associated with type 2 diabetes at genome-wide and sub-genome-wide significance were tested for replication in six independent cohorts: the Cameron County Hispanic Cohort (CCHC) [21]; the Urban American Indians and Arizona Pima Indians cohorts [22]; the Population Architecture using Genomics and Epidemiology (PAGE) study [23]; the All of Us Research Program [24]; and the Progress in Diabetes Genetics in Youth (PRODIGY), which comprises the Treatment Options for Type 2 Diabetes in Adolescents and Youth (TODAY) [25], the SEARCH for Diabetes in Youth studies [26], the Type 2 Diabetes Genetics Exploration by Next-generation sequencing in multi-Ethnic Samples (T2D-GENES) cohorts and the Mexican Metabolic Syndrome (METS) cohort [27] (ESM Table 2).

### Association with type 2 diabetes-related phenotypes

Given the lack of large-scale publicly available biobanks with Latino samples that may allow for better characterisation of our novel signals, we assembled a collection of cohorts to perform association analyses to several type 2 diabetes-related traits comprising 46 glycaemic, anthropometric and lipid traits. In addition to five of the Latino cohorts analysed in the type 2 diabetes meta-analysis (i.e. SIGMA1, SIGMA2, SIGMA3, MXBB and MGB Biobank), we included three extra cohorts, which we also imputed to the TOPMed panel: the METS and the Mexican Hypertriglyceridemia (MHTG) cohorts, as well as the genetically identified Latino samples from the UK Biobank (UKBB) [28] We also analysed the Nightingale NMR-based panel of 168 metabolomic biomarkers from the UKBB. Association analyses were done with a maximum of 26,400 adult Latino individuals, depending on the trait, of whom 19,459 were diabetes-free.

### Credible sets

For each novel variant, we identified the set of variants with 99% probability of containing the causal variant. We used a Bayesian method [29], considering variants in LD with the lead variant (*r*^2^>0.1). We calculated LD using genetic data from 1996 Hispanic/Latino samples from TOPMed freeze 5b.

### Genomic annotation

We used the 99% credible sets to annotate their genomic effect using the VEP v100 [30] (GRCh38.p7) and SNPNEXUS release Dec 2020 [31] applications. We used the Genotype–Tissue Expression project (GTEx) V8 [32] to assess the influence of the variants in gene-level expression, the TIGER Portal v7 [33] to evaluate the gene-level expression in pancreatic islets and the Islet Gene View (accessed 17 Dec 2022) [34] to assess the gene co-expression in human islets. We also assessed their association with a variety of phenotypes and diseases using the Common Metabolic Disease Knowledge Portal (cmdgenkp.org, accessed 17 Dec 2022 ) and other resources.

### Expression of genes near novel variants

We assessed the expression levels of the genes ±500 kb around the novel signals in human islets under different conditions pertaining to type 1 and type 2 diabetes. Gene expression differences between groups were assessed using *p* values and adjusted *p* values (Benjamini Hochberg correction) determined by the Wald test using the DESeq2 pipeline [35] Transcripts per million (TPM) was normalised by Salmon v1.4.0 [36].

### Polygenic scores

Polygenic scoring using single ancestry summary statistics and LD reference panels was calculated via Bayesian Regression and Continuous Shrinkage priors as implemented in PRS-CS release 4 Jun 2021 [37]. We used the UKBB LD reference panel and GWAS summary statistics from European [20], East Asian [19] and Latino populations. GWAS Latino summary statistics were calculated using a meta-analysis with five of the discovery cohorts (i.e. SIGMA1, SIGMA2, SIGMA3, MGB and GERA). Then, we used the estimated posterior SNP effect sizes for each ancestry to calculate and evaluate the performance of the polygenic scores (PSs) in a training cohort (i.e. MXBB). The best model was tested in a target cohort (i.e. the METS cohort).

Given that the ancestry-specific PSs were not highly correlated (*r*^2^<0.3), we also used PRS-CSx release 29 Jul 2021 [38], a method that improves multi-ancestry polygenic prediction by integrating GWAS summary statistics from multiple populations. We assessed the performance of the ancestry-specific vs the multi-ancestry PS.

## Results

### Overall strategy

Figure 1 summarises our overall strategy. We meta-analysed six type 2 diabetes GWAS of Latino ancestry, comprising 8150 cases and 10735 controls from hospital and population-based studies. All cohorts were imputed with TOPMed and 1000G panels and the imputation performance was evaluated. To replicate the novel loci, we analysed 13,617 type 2 diabetes cases and 20,822 controls from six independent cohorts of Latino ancestry. To gain further insight into the novel loci, we created a collection of type 2 diabetes-related phenotypes that included 26,400 Latino participants with 46 glycaemic and anthropometric traits, as well as 168 metabolomic traits. We used publicly available resources to interrogate our top signals, including functional annotation of the credible sets, and gene expression assessment of nearby genes in pancreatic islets from either type 1 or type 2 diabetes cases and controls or treated under conditions relevant for diabetes pathophysiology. We then used the generated Latino GWAS data, in combination with GWAS from other ancestries, to construct ancestry-specific and multi-ancestry type 2 diabetes PSs.

### TOPMed imputation performance

On average, imputation using the TOPMed panel resulted in 41 million (M) high-quality (*r*^2^≥0.8) variants, being 24M rare (minor allele frequency [MAF]<0.1%). This represents a 6.5-fold increased number of imputed rare variants compared with 1000G (Fig. 2a). The quality of imputation consistently improved when using TOPMed, particularly for low-frequency and rare variants (Fig. 2b).

We used WES data to confirm the improvement of TOPMed imputation to detect low-frequency and rare variants. The TOPMed panel allowed the identification of >80% of the WES variants with MAF≥0.1% compared with 60% for the same MAF cut-off with the 1000G panel (Fig. 2d). It also improved the identification of likely pathogenic variants predicted as deleterious that usually occur at low frequency (Fig. 2e).

### Type 2 diabetes GWAS meta-analysis

To illustrate the gain in discovery when using TOPMed imputation, we tested the genetic variants for association with type 2 diabetes in six Latino cohorts. Our discovery sample comprised 18,885 Latino non-related individuals (8150 cases, 10,735 controls).

**Fig. 3:**
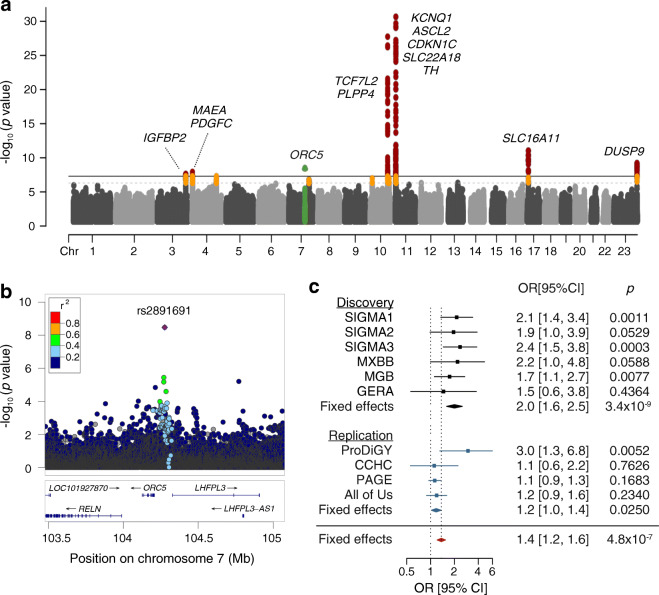
Type 2 diabetes GWAS meta-analysis in the Latino population. (**a**) Manhattan plot of the meta-analysis association statistics, highlighting the loci with genome-wide significance (red) or sub-genome-wide significance (orange) for type 2 diabetes. (**b**) Regional association plot of the novel *ORC5/LHFPL3* locus associated with type 2 diabetes risk. (**c**) Forest plot of the GWAS association statistics for the novel *ORC5/LHFPL3* locus in the discovery (black), the replication (blue) and overall (red) cohorts

We identified 26 genome-wide significant variants (*p*<5 × 10^−8^) associated with type 2 diabetes at 13 loci. Twenty-five of these were previously reported type 2 diabetes-associated variants, including those consistently identified in multiple populations (e.g. variants at *KCNQ1* and *TCF7L2*) and others enriched in the Latino population (e.g. variants at *SLC16A11*) (Fig. 3a, ESM Fig. 2 and ESM Table 3).

We identified a novel locus between the *ORC5* and *LHFPL3* genes on chromosome 7. The intergenic lead variant, rs2891691, has low frequency in Latino people and is associated with a twofold increase in the odds of developing type 2 diabetes in the discovery sample (MAF 1.7%; OR 2.0 [95% CI 1.59, 2.52], *p*=3.4 × 10^−9^) (Fig. 3b,c). Although it was also imputed with the 1000G panel, TOPMed’s higher imputation quality strengthened the association (1000G, mean ± SD imputation *r*^2^=0.948 ± 0.057, *p*=2.3 × 10^−8^; TOPMed, mean ± SD imputation *r*^2^=0.983 ± 0.009, *p*=3.4 × 10^−9^).

This variant is rare in Europeans (MAF 0.04%), yet prevalent among African (MAF 16%) and East Asian populations (MAF 7.6%). However, its association with type 2 diabetes does not replicate in either Africans (*p*=0.149) or East Asians (*p*=0.095). A fixed effects meta-analysis of the three ancestries showed no association of the variant with type 2 diabetes (*p*=0.734) but a significant heterogeneity in the allelic effects (*p*=5 × 10^−8^). To further investigate the source of such heterogeneity, we used MR-MEGA v1.0.5 software [39], which implements a multi-ancestry meta-regression approach to model allelic effects as a function of axes of the genetic variation. This meta-regression approach showed a significant association of rs2891691 with type 2 diabetes (*p*=1.1 × 10^−7^), as well as significant heterogeneity of the allelic effects between populations driven by ancestry (*p*=2.9 × 10^−8^). The residual heterogeneity accounting for other factors, such as phenotype definition or uncorrected population structure, was not significant (*p*=0.944) (ESM Fig. 3). These results show that the effects of rs2891691 on type 2 diabetes are specific to the Latino population and suggest that the lead variant we identified is in LD with the causal variant in Latino but not African or East Asian populations, a phenomenon also observed in a previous type 2 diabetes multi-ancestry meta-analysis [40] The heterogeneity in the allelic effects across ancestries can also be explained by differences in environmental exposures.

A sex-dimorphism in *RELN* gene expression has been documented, with higher *RELN* expression in women [41] and sex hormones likely mediating *RELN* expression. Because of the proximity of *RELN* to rs2891691, we evaluated the sex-specific association with type 2 diabetes and tested for heterogeneity between sex-specific allelic effects using GWAMA v2.2.2 [42]. rs2891691 showed a larger effect and was more associated with type 2 diabetes in women (Neff 10,228; OR 2.4 [95% CI 1.73, 3.22], *p*=6.6 × 10^−8^) compared with men (Neff 7206; OR 1.5 [95% CI 1.08, 2.19], *p*=0.018), yet the between-sex heterogeneity did not reach statistical significance (*p*=0.076) (ESM Table 4).

### Replication analysis

The replication analysis comprised 13,617 type 2 diabetes cases and 20,822 controls (ESM Table 2). The meta-analysis of the replication cohorts, where the variant was present, was nominally significant and showed a consistent direction of effect with the discovery sample (OR 1.18 [95% CI 1.02, 1.36], *p*=0.025) (Fig. 3c, ESM Table 5).

By querying our Latino collection of type 2 diabetes-related phenotypes, we found that the rs2891691 risk allele C was nominally associated with lower fasting glucose levels (*p*=0.026) (ESM Table 6). Such negative correlation might be induced by collider bias since specifically for glycaemic traits we only analysed diabetes-free individuals. Indeed, a positive association of rs2891691 risk allele with 2 h glucose adjusted for BMI has been previously reported in Latino ancestry participants (β=3.4 mg/dl [0.2 mmol/l], *p*=0.006) [43] and low potassium levels in East Asian ancestry participants (*p*=8.5 × 10^−5^) [44]. Accumulated epidemiological evidence points to a relationship between low potassium levels and decreased insulin secretion and risk of type 2 diabetes [45, 46]

The 99% credible set consisted only of the lead variant rs2891691 (ESM Table 7), yet we cannot discard other variants not called due to genotyping complexity nor those imputed to the TOPMed panel, such as a structural, variable tandem repeat or copy number variants.

To better characterise the role of the *ORC5*/*LHFPL3* locus, we assessed gene expression using the GTEx [32] and TIGER [33] portals. *ORC5* is expressed ubiquitously, while *LHFPL3* is specifically expressed in the brain (ESM Fig. 5a, b). We then assessed the expression levels of genes ±500 kb around the novel signal in human islets under different conditions relevant to diabetes pathophysiology. *ORC5* was downregulated after 2 h and 8 h exposure to IFN-α, and upregulated by exposure to brefeldin A (ESM Fig. 6a, c). Both IFN-α and brefeldin A are endoplasmic reticulum stress inducers that reduce the insulin content with a rise in the proinsulin/insulin ratio [47] and inhibit glucose-stimulated insulin secretion [48], respectively.

### Prioritising sub-genome-wide significant variants

**Fig. 4:**
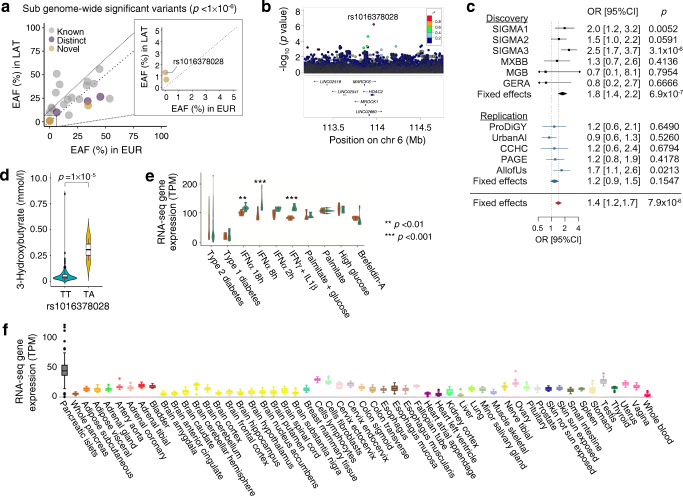
Sub-genome-wide significant *HDAC2* novel type 2 diabetes loci. (**a**) Scatter plot of the effect allele frequencies (EAFs) from the sub-genome-wide significant variants in Latino (LAT) vs European (EUR) populations, highlighting those that are distinct from the known lead type 2 diabetes-associated variants (purple) and those that are in novel loci (yellow). (**b**) Regional association plot of the novel *HDAC2* locus associated with type 2 diabetes risk. (**c**) Forest plot of the association statistics in the discovery (black) and the replication (blue) cohorts. (**d**) Violin plots of serum 3-hydroxybutyrate levels in non-carriers (blue) and carriers (yellow) of the rs1016378028 variant. Whiskers range from upper and lower fences (1.5 × IQR); points represent outliers. (**e**) *HDAC2* gene expression in human islets from donors with type 1 and type 2 diabetes and control islets treated (brown) or not (green) with different cytokines or other stressor compounds. **p<0.01, ***p<0.001 vs no treatment (adjusted *p* values, Benjamini–Hochberg correction). Whiskers range from upper and lower fences (1.5 × IQR); points represent outliers. (**f**) *HDAC2* gene expression in multiple tissues from GTEx and TIGER portals. Each box plot shows expression in a different tissue or cell line Whiskers range from upper and lower fences (1.5 × IQR); points represent outliers

We next searched for variants that were associated with type 2 diabetes at sub-genome-wide significance (*p*<5 × 10^−6^) but that deserved further study as they lay in previously unreported type 2 diabetes loci, were enriched or Latino-specific, and/or exclusively imputed with the TOPMed panel (Fig. 4a and ESM Table 8). Three out of the 23 sub-genome-wide lead variants lay in or near the known type 2 diabetes loci *TACC2*, *FGFR2* and *CCND2.* We considered them as distinct variants as they retained locus-wide significance (*p*<5 × 10^−5^) after conditioning on the nearest known associated variant.

Three additional sub-genome-wide significant variants were located ±1 Mb away from any reported type 2 diabetes locus (Fig. 4a and ESM Table 9). Of interest, rs1016378028 is a low-frequency variant (MAF 1.3%; OR 1.77 [95% CI 1.41, 2.21], *p*=7.0 × 10^−7^) that is Latino private (MAF<0.01% in other populations) and is only imputed with the TOPMed panel. It is intronic of *HDAC2*, a gene under strong purifying selection (probability of being LoF intolerant [pLI]=1, gnomAD, gnomAD-sg.org, accessed 17 December 2022) and that is highly and mostly expressed in pancreatic islets (tiger.bsc.es, accessed 17 December 2022) (Fig. 4f) [33].

Although the replication results did not show statistical significance, the direction of the effect was consistent with the discovery effect (OR 1.17 [95% CI 0.94, 1.45], *p*=0.1547) (Fig. 4b,c, ESM Table 5). The Diabetes Meta-Analysis of Trans-Ethnic association studies (DIAMANTE) European meta-analysis [20] reported a suggestive signal ~80 kb upstream of rs1016378028 (rs4945979, *p*=4.8 × 10^−6^). After conditioning for the rs4945979 variant, the statistical significance of our identified variant remained essentially the same (OR 1.75 [95% CI 1.4, 2.2], *p*=4.5 × 10^−7^).

The rs1016378028 risk allele was significantly associated with higher levels of acetone (*p*=1.2 × 10^−7^), 3-hydroxybutyrate (*p*=1.01 × 10^−5^) and acetoacetate (*p*=3.3 × 10^−5^) (Fig. 4d and ESM Table 10). It was also nominally associated with lower hip circumference (*p*=0.02) and higher WHR (*p*=0.03) (ESM Table 6).

*HDAC2* expression in human islets is downregulated after exposure to IFN-α (8 h log_2_-fold change=−0.38, *p*=6 × 10^−7^; 18 h log_2_-fold change=−0.28, *p*=3 × 10^−4^) or IFN-γ+IL-1β (log_2_-fold change=−0.39, *p*=3 × 10^−7^) (Fig. 4e). These cytokines mimic the proinflammatory milieu of type 1 diabetes, inhibit beta cell function [49, 50], induce beta cell stress and may trigger beta cell dedifferentiation in type 2 diabetes [51, 52].

### Development of PSs for the Latino population

**Fig. 5:**
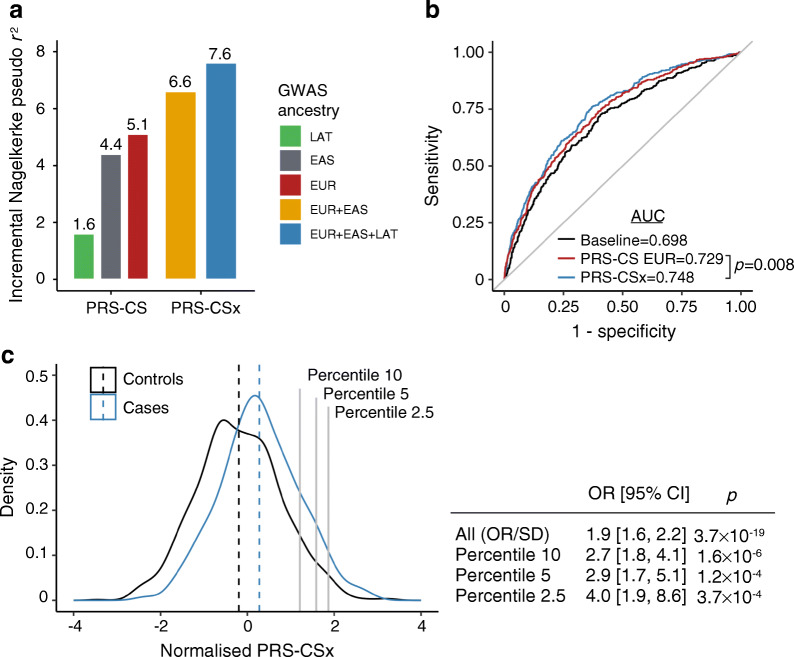
PS for the risk of type 2 diabetes in Latino population. (**a**) Variance explained by a PS using these Latino GWAS association statistics (LAT, green), the AGEN East Asian GWAS association statistics (EAS, grey), the DIAMANTE European GWAS association statistics (EUR, red), a combination of DIAMANTE European and AGEN East Asian GWAS association statistics (yellow) and a combination of DIAMANTE European, AGEN East Asian and these Latino GWAS association statistics (blue). METSB was used as the testing cohort. (**b**) Receiver operating characteristic curves for the type 2 diabetes risk prediction explained by a model including sex, age and ten PCs of ancestry (black), a model including covariates and a PS constructed using DIAMANTE European (red) and a model including covariates and a PS constructed using a combination of DIAMANTE European, AGEN East Asian and these Latino GWAS association statistics (blue). (**c**) Distribution of a multi-ancestry PS using a combination of DIAMANTE European, AGEN East Asian and these Latino GWAS association statistics in type 2 diabetes cases (blue) and controls (black). The table shows the OR per SD attributed to the multi-ancestry PS, as well as the OR for high-risk individuals

We then developed a PS for type 2 diabetes in Latino people using our TOPMed imputed GWAS meta-analysis data. This PS explained 1.6% of the type 2 diabetes status variance (Fig. 5a), which is expected given the relatively small sample size of the Latino summary statistics compared with European and East Asian ancestries. The PS derived from the Diabetes Meta-Analysis of Trans-Ethnic association studies (DIAMANTE) European GWAS [20] and from Asian Genetic Epidemiology Network (AGEN) East Asian GWAS [19] explained 5.1% and 4.4% of the type 2 diabetes variance in the Latino population, respectively. The European and East Asian PSs showed a weak correlation (*r*^2^<0.2) with our Latino TOPMed-derived PS, suggesting that they could provide orthogonal information and improve the overall predictive performance. We developed a PS that incorporated GWAS data from the three ancestries using PRS-CSx [38], a method that allows for the integration of summary statistics and LD reference panels from different ancestries. The multi-ancestry PS including the three GWAS summary statistics explained 7.6% of the type 2 diabetes variance in the Latino target sample. Our Latino GWAS added 1% of the explained variance compared with the PS using only European and East Asian GWAS, which explained 6.6% of the variance.

Each SD of the multi-ancestry PS was associated with an OR of 1.9 (95% CI 1.6, 2.2, *p*=3.7 × 10^−19^) (Fig. 5c). People in the 2.5 percentile of the PS showed four times more risk of developing type 2 diabetes (OR 4.01 [95% CI 1.87, 8.62], *p*=3.7 × 10^−4^) (Fig. 5c). The receiver operating characteristic AUC of the full model including the multi-ancestry PS was 0.748 (95% CI 0.72, 0.775) compared with 0.729 (95% CI 0.701, 0.758) of the PS including European GWAS only, representing a 2% improvement in the prediction accuracy (*p*=0.008) (Fig. 5b).

## Discussion

The Latino population has been underrepresented in most genetic studies. Yet, recent studies of type 2 diabetes in Latino populations have been fruitful, even with sample-size orders of magnitude smaller than those in studies of European or East Asian ancestries. The poor representation of Latino samples with genotype and phenotype data constrains nearly every step of a gene–disease association framework, including genotype imputation, a cost-effective technique to improve the resolution of a GWAS. This is more problematic for low-frequency and rare variation. Instead, next-generation sequencing technologies have typically been chosen but these are more expensive, precluding the study of large samples. This study was motivated by the recent release of the TOPMed imputation panel, which includes the largest number of Latino haplotypes compared with all available panels.

In this study, we aggregate genotype and WES data from six datasets to test the improvement in accuracy of the TOPMed imputation compared with 1000G. To illustrate how this panel can boost the discovery of complex disease variants we performed a type 2 diabetes GWAS meta-analysis using the imputed data. TOPMed imputation not only improved the statistical significance of our findings but allowed for the testing of up to 24 M rare variants, compared with 3 M properly imputed with the 1000G panel. The high quality of TOPMed imputation at low/rare frequencies is especially relevant for the study of disease-causing variation, because deleterious variants usually span such a spectrum. We show that by imputing with TOPMed, it is possible to test >90% of the variants with a MAF≥0.1% predicted to be deleterious by the Combined Annotation Dependent Depletion (CADD) score; previously, it was only possible to detect these variants by relying on more expensive sequencing technologies. While ascertaining variants at frequencies <0.1% may still require whole-genome sequencing (WGS) or WES, we estimate that the power to identify associated variants may be limited unless we undertake sequencing efforts with sample sizes orders of magnitude larger than our study. For example, for MAF<0.1%, the effective sample size required to reach statistical power to detect associations with an effect of OR>2.0 is above 170,000 individuals (Fig. 2c). Since the cost of sequencing such a large sample size is a major constraint for the study of underrepresented populations, we propose that highly accurate imputation with dense reference panels may be a more cost-effective approach.

In this study, we identified a novel low-frequency variant associated with type 2 diabetes, rs2891691, which lies between the *ORC5* and *LHFPL3* genes and showed increased accuracy of imputation and association power when using the TOPMed panel. *ORC5* encodes the subunit 5 of the origin recognition complex implicated in the DNA replication origins, transcription silencing and heterochromatin formation [53] Lipoma HMGIC fusion partner-like 3 (LHFPL3) is a member of the tetraspanin superfamily, which functions as membrane protein organiser. The rs2891691 risk allele is present in 1% of Latino people. Overall, in discovery and replication cohorts, carriers have 1.37-fold increased odds of developing type 2 diabetes, with a possibly higher risk in women.

We identified a second low-frequency variant, rs1016378028, associated with a 1.7-fold increased risk of type 2 diabetes, which is not imputed with the 1000G panel. This variant was prioritised from a subset of variants at a sub-genome-wide significant threshold that showed additional evidence of association. rs1016378028 is a Latino private variant (MAF: Latino, 1.3%; East Asian, 0.2%; other populations, <0.05%), and lies within *HDAC2*, a gene that is highly intolerant of protein-changing variation and is mostly expressed in pancreatic islets [33].

Histone deacetylase 2 (HDAC2) is a histone deacetylase involved in gene transcription repression. HDACs play a regulatory role in insulin signalling, beta cell function and pancreatic endocrine cell development. At low glucose levels, HDAC2 is recruited to the insulin promoter to downregulate its expression [54]. In human islets, *HDAC2* expression negatively correlates with insulin gene expression (*r*=−0.56, false discovery rate 3.7 × 10^−16^) and positively correlates with *IAPP* expression, which encodes for a satiety hormone (*r*=0.38, false discovery rate 1.8 × 10^−7^) [34] HDAC2 also deacetylates IRS-1, uncoupling its downstream phosphorylation cascade. Both insulin expression and insulin signalling are partially restored after treatment with HDAC2 inhibitors [55, 56]. We show that cytokine treatment of pancreatic islets downregulated *HDAC2* expression.

Because there are no comprehensive phenome-wide association data to guide the interpretation of variants enriched in Latino populations, we aggregated phenotypic glycaemic and cardiometabolic data from 26,400 Latino individuals to follow-up the identified variants. We found that rs1016378028 risk allele carriers have higher levels of ketone bodies, which are produced through the breakdown of fatty acids and serve as an alternative energy source to glucose. Uncoupled hepatic production of ketone bodies may be a pathological consequence of relative insulin deficiency in diabetes [57]. While the mechanism linking rs1016378028, diabetes and 3-hydroxybutyrate levels remains to be determined, our results suggest this variant as a potential genetic type 2 diabetes risk factor.

We leveraged our GWAS results and existing publicly available data to develop an improved PS for Latino ancestry. PSs developed in a particular ancestry group poorly transfer to other populations, exacerbating disparities between populations. We provide an improved PS for the Latino population, by using a combination of GWAS and LD data from East Asian, European and our Latino GWAS. This PS showed a similar performance to the previously reported in European ancestry [58] with individuals at the top 2.5 percentile showing a fourfold increased risk of type 2 diabetes. Evaluating this PS in additional external datasets of Latino ancestry may prove useful in assessing its potential clinical utility.

Leveraging new resources to reanalyse Latino data, such as imputation with the TOPMed panel, proved to be successful in identifying additional type 2 diabetes-related loci. We acknowledge that the TOPMed panel allows the testing of an increased number of variants and additional evidence will be needed to confirm associations at the standard GWAS significance. Further efforts are needed to increase the power of discovery and to follow-up on novel findings in diverse populations. Until then, translation of identified genetic variation-to-function and application to the clinic in Latino populations will remain highly compromised compared with the resources available for European populations. In this study we gathered a high number of Latino samples with extensive biomarker and clinical characterisation; however, larger sample sizes are still needed to achieve sufficient statistical power to detect low-frequency variants. Efforts must be expanded to build shareable resources with a high representation of different ancestries, enabling ancestry-specific effects to be interpreted within the local ancestry context, which is instrumental to identify causal genes, to improve the biological mechanistic insight and to develop targeted therapies.

Overall, this study confirms the superior imputation performance of TOPMed, representing a cost-effective and unique opportunity to analyse low-frequency and rare genetic variants in Latino samples at scale. It also presents the largest type 2 diabetes GWAS meta-analysis performed in individuals of Latino ancestry imputed with the TOPMed reference panel. Despite the sample size being orders of magnitude smaller compared with studies performed in other populations, the novel discoveries presented here suggest that more novel genetic associations and new biology of type 2 diabetes will be revealed as the sample size of discovery samples, reference panels and large-scale biobanks with phenome-wide data increase in studies including non-European populations.

## Supplementary information

ESM 1 (PDF 1096 kb)

ESM 2 (XLSX 109 kb)

## Data Availability

Full summary statistics are available through the Common Metabolic Diseases Knowledge Portal (https://t2d.hugeamp.org/downloads.html) and through the GWAS catalog (https://www.ebi.ac.uk/gwas/, accession ID: GCST90255648). Polygenic scores (PS) weights for each ancestry are available via the PGS catalog (https://www.pgscatalog.org, publication ID: PGP000445, scores IDs: PGS003443, PGS003444 and PGS003445).

## Abbreviations

1000G: 1000 Genomes
AGEN: Asian Genetic Epidemiology Network
AF: Allele frequency
CADD: Combined Annotation Dependent Depletion
CCHC: Cameron County Hispanic Cohort
DIAMANTE: Diabetes Meta-Analysis of Trans-Ethnic association studies
FNRS: Fonds National de la Recherche Scientifique
GERA: Genetic Epidemiology Research on Aging
GTEx: Genotype–Tissue Expression project
GWAS: Genome-wide association study
LD: Linkage disequilibrium
LoF: Loss-of-function
MAF: Minor allele frequency
METS: Mexican Metabolic Syndrome (cohort)
MGB: Mass General Brigham Biobank
MHTG: Mexican Hypertriglyceridemia
MXBB: Mexican Biobank
Neff: Effective sample size
NHGRI: National Human Genome Research Institute
NHLBI: National Heart, Lung, and Blood Institute
NIMHD: National Institute on Minority Health and Health Disparities
PAGE: Population Architecture using Genomics and Epidemiology
PC: Principal component
PRODIGY: Progress in Diabetes Genetics in Youth
PS: Polygenic score
SIGMA: Slim Initiative for Genomic Medicine in the Americas
T2D-GENES: Type 2 Diabetes Genetics Exploration by Next-generation sequencing in multi-Ethnic Samples
TODAY: Treatment Options for Type 2 Diabetes in Adolescents and Youth
TOPMed: NHLBI Trans-Omics for Precision Medicine
TPM: Transcripts per million
UKBB: UK Biobank
WES: Whole-exome sequencing

## References

[1] Williams Amy AL, Jacobs Suzanne SBR, Moreno-Macías H (2014). Sequence variants in SLC16A11 are a common risk factor for type 2 diabetes in Mexico. Nature.

[2] Estrada K, Aukrust I, The SIGMA Type 2 Diabetes Consortium (2014). Association of a low-frequency variant in HNF1A with type 2 diabetes in a Latino population. JAMA.

[3] Mercader JM, Liao RG, Bell AD (2017). A loss-of-function splice acceptor variant in IGF2 is protective for type 2 diabetes. Diabetes.

[4] Rusu V, Rusu V, Hoch E (2017). Type 2 diabetes variants disrupt function of SLC16A11 through two distinct mechanisms. Cell.

[5] Mercader JM, Florez JC (2017). The genetic basis of type 2 diabetes in Hispanics and Latin Americans: challenges and opportunities. Front Public Health.

[6] Das S, Abecasis GR, Browning BL (2018). Genotype imputation from large reference panels. Annu Rev Genomics Hum Genet.

[7] Auton A, Abecasis GR, Altshuler DM (2015). A global reference for human genetic variation. Nature.

[8] Kowalski MH, Qian H, Hou Z (2019). Use of >100,000 NHLBI Trans-Omics for Precision Medicine (TOPMed) Consortium whole genome sequences improves imputation quality and detection of rare variant associations in admixed African and Hispanic/Latino populations. PLoS Genet.

[9] Taliun D, Harris DN, Kessler MD (2021). Sequencing of 53,831 diverse genomes from the NHLBI TOPMed Program. Nature.

[10] Sepúlveda J, Tapia-Conyer R, Velásquez O et al (2007) Diseño y metodología de la Encuesta Nacional de Salud 2000. Salud Publica Mex 49(Suppl 3):427–432 [article in Spanish]

[11] Karlson EW, Boutin NT, Hoffnagle AG, Allen NL (2016). Building the Partners Healthcare Biobank at Partners Personalized Medicine: informed consent, return of research results, recruitment lessons and operational considerations. J Pers Med.

[12] Banda Y, Kvale MN, Hoffmann TJ (2015). Characterizing race/ethnicity and genetic ancestry for 100,000 subjects in the Genetic Epidemiology Research On Adult Health And Aging (GERA) cohort. Genetics.

[13] Alexander DH, Novembre J, Lange K (2009). Fast model-based estimation of ancestry in unrelated individuals. Genome Res.

[14] Delaneau O, Marchini J, Zagury JF (2012). A linear complexity phasing method for thousands of genomes. Nat Methods.

[15] Sudmant PH, Rausch T, Gardner EJ (2015). An integrated map of structural variation in 2,504 human genomes. Nature.

[16] Cingolani P, Platts A, Wang LL (2012). A program for annotating and predicting the effects of single nucleotide polymorphisms, SnpEff: SNPs in the genome of Drosophila melanogaster strain w1118; iso-2; iso-3. Fly (Austin).

[17] Marchini J, Howie B, Myers S, McVean G, Donnelly P (2007). A new multipoint method for genome-wide association studies by imputation of genotypes. Nat Genet.

[18] Willer CJ, Li Y, Abecasis GR (2010). METAL: Fast and efficient meta-analysis of genomewide association scans. Bioinformatics.

[19] Spracklen CN, Horikoshi M, Kim YJ (2020). Identification of type 2 diabetes loci in 433,540 East Asian individuals. Nature.

[20] Mahajan A, Taliun D, Thurner M (2018). Fine-mapping type 2 diabetes loci to single-variant resolution using high-density imputation and islet-specific epigenome maps. Nat Genet.

[21] Fisher-Hoch SP, Rentfro AR, Salinas JJ (2010). Socioeconomic status and prevalence of obesity and diabetes in a Mexican American community, Cameron County, Texas, 2004-2007. Prev Chronic Dis.

[22] Nair AK, Sutherland JR, Traurig M (2018). Functional and association analysis of an Amerindian-derived population-specific p.(Thr280Met) variant in RBPJL, a component of the PTF1 complex. Eur J Hum Genet.

[23] Wojcik GL, Graff M, Nishimura KK (2019). Genetic analyses of diverse populations improves discovery for complex traits. Nature.

[24] Denny J, Rutter JL, All of Us Research Program Investigators (2019). The “All of Us” research program. N Engl J Med.

[25] Haymond M, Anderson B, Barrera P (2007). Treatment options for type 2 diabetes in adolescents and youth: a study of the comparative efficacy of metformin alone or in combination with rosiglitazone or lifestyle intervention in adolescents with type 2 diabetes. Pediatr Diabetes.

[26] SEARCH Study Group (2004). SEARCH for Diabetes in Youth: a multicenter study of the prevalence, incidence and classification of diabetes mellitus in youth. Control Clin Trials.

[27] Arellano-Campos O, Gómez-Velasco DV, Bello-Chavolla OY (2019). Development and validation of a predictive model for incident type 2 diabetes in middle-aged Mexican adults: The metabolic syndrome cohort. BMC Endocr Disord.

[28] Ahola-Olli AV, Mustelin L, Kalimeri M (2019). Circulating metabolites and the risk of type 2 diabetes: a prospective study of 11,896 young adults from four Finnish cohorts. Diabetologia.

[29] Maller JB, McVean G, The Wellcome Trust Case Control Consortium (2012). Bayesian refinement of association signals for 14 loci in 3 common diseases. Nat Genet.

[30] McLaren W, Gil L, Hunt SE (2016). The ensembl variant effect predictor. Genome Biol.

[31] Oscanoa J, Sivapalan L, Gadaleta E, Dayem Ullah AZ, Lemoine NR, Chelala C (2020). SNPnexus: a web server for functional annotation of human genome sequence variation (2020 update). Nucleic Acids Res.

[32] GTEx Consortium (2013). The Genotype-Tissue Expression (GTEx) project. Nat Genet.

[33] Alonso L, Piron A, Morán I (2021). TIGER: The gene expression regulatory variation landscape of human pancreatic islets. Cell Rep.

[34] Asplund O, Storm P, Chandra V (2022). Islet gene view—a tool to facilitate islet research. Life Sci Alliance.

[35] Love MI, Huber W, Anders S (2014). Moderated estimation of fold change and dispersion for RNA-seq data with DESeq2. Genome Biol.

[36] Patro R, Duggal G, Love MI, Irizarry RA, Kingsford C (2017). Salmon provides fast and bias-aware quantification of transcript expression. Nature Methods.

[37] Ge T, Chen CY, Ni Y, Feng YCA, Smoller JW (2019). Polygenic prediction via Bayesian regression and continuous shrinkage priors. Nat Commun.

[38] Ruan Y, Lin Y-F, Feng Y-CA (2022). Improving polygenic prediction in ancestrally diverse populations. Nat Genet.

[39] Mägi R, Horikoshi M, Sofer T (2017). Trans-ethnic meta-regression of genome-wide association studies accounting for ancestry increases power for discovery and improves fine-mapping resolution. Hum Mol Genet.

[40] Kanai M, Ulirsch JC, Karjalainen J et al (2021) Insights from complex trait fine-mapping across diverse populations. medRxiv 2021.09.03.21262975. 10.1101/2021.09.03.21262975

[41] Eastwood SL, Harrison PJ (2003). Interstitial white matter neurons express less reelin and are abnormally distributed in schizophrenia: towards an integration of molecular and morphologic aspects of the neurodevelopmental hypothesis. Mol Psychiatry.

[42] Magi R, Lindgren CM, Morris AP (2010). Meta-analysis of sex-specific genome-wide association studies. Genet Epidemiol.

[43] Chen J, Spracklen CN, Marenne G (2021). The trans-ancestral genomic architecture of glycemic traits. Nat Genet.

[44] Kanai M, Akiyama M, Takahashi A (2018). Genetic analysis of quantitative traits in the Japanese population links cell types to complex human diseases. Nat Genet.

[45] Ekmekcioglu C, Elmadfa I, Meyer AL, Moeslinger T (2016). The role of dietary potassium in hypertension and diabetes. J Physiol Biochem.

[46] Heianza Y, Hara S, Arase Y (2011). Low serum potassium levels and risk of type 2 diabetes: the Toranomon Hospital Health Management Center Study 1 (TOPICS 1). Diabetologia.

[47] Lombardi A, Tomer Y (2017) Interferon alpha impairs insulin production in human beta cells via endoplasmic reticulum stress. J Autoimmun 80:48–55. 10.1016/J.JAUT.2017.02.002 10.1016/j.jaut.2017.02.002 PMC5758382 28238527

[48] Bone R, Oyebamiji O, Talware S (2020). A computational approach for defining a signature of β-cell Golgi stress in diabetes. Diabetes.

[49] Eizirik DL, Pasquali L, Cnop M (2020). Pancreatic β-cells in type 1 and type 2 diabetes mellitus: different pathways to failure. Nat Rev Endocrinol.

[50] Marroqui L, dos Santos RS, Op de Beeck A (2017). Interferon-α mediates human beta cell HLA class I overexpression, endoplasmic reticulum stress and apoptosis, three hallmarks of early human type 1 diabetes. Diabetologia.

[51] Oshima M, Knoch KP, Diedisheim M (2018). Virus-like infection induces human β cell dedifferentiation. JCI Insight.

[52] Sun J, Ni Q, Xie J (2019). β-Cell dedifferentiation in patients with T2D with adequate glucose control and nondiabetic chronic pancreatitis. J Clin Endocrinol Metab.

[53] Shen Z (2013). The origin recognition complex in human diseases. Biosci Rep.

[54] Mosley AL, Özcan S (2004). The pancreatic duodenal homeobox-1 protein (PDX-1) interacts with histone deacetylases HDAC-1 and HDAC-2 on low levels of glucose. J Biol Chem.

[55] Christensen DP, Dahllöf M, Lundh M (2011). Histone deacetylase (HDAC) inhibition as a novel treatment for diabetes mellitus. Mol Med.

[56] Ye J (2013). Improving insulin sensitivity with HDAC inhibitor. Diabetes.

[57] Longo VD, Mattson MP (2014). Fasting: molecular mechanisms and clinical applications. Cell Metab.

[58] Khera AV, Chaffin M, Aragam KG (2018). Genome-wide polygenic scores for common diseases identify individuals with risk equivalent to monogenic mutations. Nat Genet.

